# Chemical Constituents from Leaves of *Baccharis sphenophylla* (Asteraceae) and Their Antioxidant Effects

**DOI:** 10.3390/plants12061262

**Published:** 2023-03-10

**Authors:** Marcela H. Retamozo, Christian C. Silva, Cinthia I. Tamayose, Juliana C. S. Carvalho, Paulete Romoff, Oriana A. Fávero, Marcelo J. P. Ferreira

**Affiliations:** 1 Departamento de Botânica, Instituto de Biociências, Universidade de São Paulo, São Paulo 05508-090, SP, Brazil; 2 São Bernardo College, São Bernardo do Campo 09715-020, SP, Brazil; 3 Universidade Presbiteriana Mackenzie, São Paulo 01302-907, SP, Brazil

**Keywords:** *Baccharis sphenophylla*, Compositae, flavonoids, chlorogenic acid derivatives, antiradical activity

## Abstract

*Baccharis* is one of the largest genera of Asteraceae and its species are used in folk medicine for several medicinal purposes due to the presence of bioactive compounds. We investigated the phytochemical composition of polar extracts of *B. sphenophylla*. Using chromatographic procedures, diterpenoids (*ent*-kaurenoic acid), flavonoids (hispidulin, eupafolin, isoquercitrin, quercitrin, biorobin, rutin, and vicenin-2), caffeic acid, and chlorogenic acid derivatives (5-*O*-caffeoylquinic acid and its methyl ester, 3,4-di-*O*-caffeoylquinic acid, 4,5-di-*O*-caffeoylquinic acid, and 3,5-di-*O*-caffeoylquinic acid and its methyl ester) were isolated from polar fractions and are described. The extract, polar fractions, and fifteen isolated compounds were evaluated in relation to radical scavenging activity using two assays. Chlorogenic acid derivatives and flavonols exhibited higher antioxidant effects, confirming that *B. sphenophylla* is an important source of phenolic compounds with antiradical properties.

## 1. Introduction

*Baccharis* L. is one of the largest and most highly diversified genera of Asteraceae found in the New World. The number of species recognized within the genus ranges from 354 to ca. 500 species [1,2]. Approximately 90% of *Baccharis* species are found in South America, and they are distributed mainly in the warm temperate and tropical regions of Argentina, Brazil, Chile, Colombia, and Mexico. In Brazil, 179 species have been described and, among them, 114 are endemic species [3].

Several *Baccharis* species are used in folk medicine for various medicinal purposes: as analgesic, diuretic, and hepatoprotective medicines; for the treatment of diabetes, fever, and gastrointestinal illnesses; and for detoxification and control of obesity, as manifest in the form of the widespread and popular use of “carqueja” (*Baccharis crispa* Spreng.; syn: *B. trimera* (Less.) DC.) as a slimming tea [4,5,6,7,8]. Moreover, various biological activities have also been described, including antibacterial, antifungal, antiviral, antiprotozoal, anti-inflammatory, cytotoxic, and antioxidant activities [4,5,6,7,8,9,10,11,12,13,14,15,16,17,18,19]. Additionally, the genus is known for its aromatic properties and is widely used in the cosmetic and herbal industries. Previous phytochemical studies of the genus reported several classes of natural products, such as cinnamic acid derivatives [7,8,9,10,11], flavonoids [10,11,12,13,14,15,16], terpenoids [11,15,16], and volatile oils [17,18,19,20,21,22,23].

**Figure 1:**
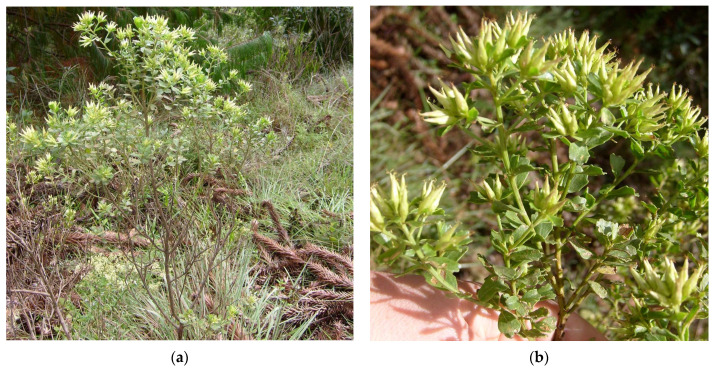
Photos of *Baccharis sphenophylla* in the field. (**a**) Photo of an entire specimen of *B. sphenophylla* in the field; (**b**) zoomed-in photo of the leaves and flower buds of *B. sphenophylla*.

In our continuing efforts to search for bioactive compounds in *Baccharis* species [24,25], we have focused on *B. sphenophylla* Dusén ex Malme (Figure 1), a rare and endemic plant found in the highlands of south and southeast Brazil; specifically, in the states of Paraná, São Paulo, and Minas Gerais. Previously, the *n*-hexane extract of this species was studied, demonstrating a long-chain ester of *p*-coumaric acid, two sesquiterpenoids, and four diterpenoids with antitrypanosomal effects [26]. Herein, we describe the isolation, structure, and antiradical activities of compounds obtained from polar extracts of the leaves of *B. sphenophylla*.

## 2. Results

### 2.1. Structural Elucidation of Compounds

**Figure 2:**
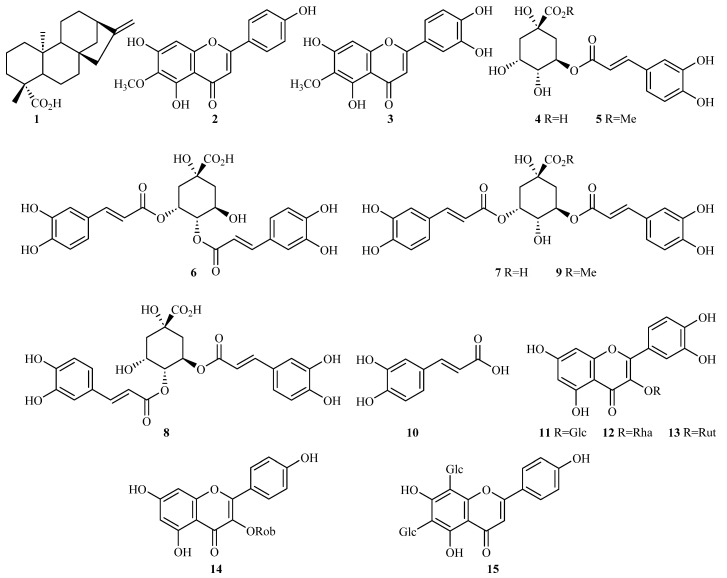
Structures of compounds identified from leaves of *B. sphenophylla*.

Extracts from leaves of *Baccharis sphenophylla* (Asteraceae) were obtained with hexane and, subsequently, with ethanol, both until exhaustion. The ethanol extract was partitioned with dichloromethane (DCM), ethyl acetate (EtOAc), and *n*-butanol (BuOH). The chromatographic fractionation of the DCM fraction allowed the isolation of compounds **1–3** (Figure 2). Compound **1** exhibited both ^1^H and ^13^C NMR data compatible with the *ent*-kaurenoic acid, a diterpenoid previously isolated from *B. sphenophylla* [26]. Compounds **2** and **3** showed singlets at δ 6.80 and δ 6.66 in their ^1^H NMR spectra, respectively, which were assigned to H-3 from a flavone skeleton. These data were corroborated by the UV spectra, which showed absorption bands at 269 nm and 335 nm and at 269 nm and 345 nm, respectively, both characteristic of flavones [27]. Compound **2** showed two doublets at δ 7.89 (d, *J* = 8.8 Hz, 2H) and δ 6.92 (d, *J* = 8.8 Hz, 2H) corresponding to *ortho*-coupling between H-2′,6′/H-3′,5′ from the 4′-hydroxylated ring B of flavones. On the other hand, compound **3** showed a typical 3’,4´-dioxygenated substitution pattern at ring B, exhibiting two doublets at δ 7.40 (d, *J* = 2.0 Hz, 1H) and δ 6.89 (d, *J* = 8.0 Hz, 1H) and a doublet of doublets at δ 7.42 (dd, *J* = 8.0, 2.0 Hz, 1H). Both compounds showed a singlet at δ 6.58, which was assigned to H-8 of the 5,6,7-trioxygenated A-ring, and a methoxyl group was inferred from a singlet at δ 3.75 (s, 3H). Through heteronuclear multiple bond correlations (HMBCs), these groups were bonded at C-6 in both compounds. These data were consistent with the structures of the flavonoids hispidulin (**2**) and eupafolin (**3**) (Figure 2), which was subsequently confirmed through a comparison of the spectroscopic data (^1^H and ^13^C NMR) with those reported in the literature [28,29].

Chromatographic procedures carried out with the EtOAc fraction yielded the isolation of compounds **4**–**14**. Compounds **4**–**9** showed two doublets in their spectra found in the regions δ 7.62–7.52 (d, *J* = 15.9 Hz, 1H) and δ 6.36–6.19 (d, *J* = 15.9 Hz, 1H), which were attributed to *trans*-hydrogens from α,β-unsaturated carboxyl groups; two doublets at 7.07–7.01 (d, *J* = 1.9 Hz, 1H) and 6.80–6.73 (d, *J* = 8.2 Hz, 1 H) corresponding to *meta*- and *ortho*-couplings between hydrogens and assigned to H-2′ and H-5′, respectively; and a doublet of doublets at 6.97–6.88 (dd, *J* = 8.2, 1.9 Hz, 1H) assigned to H-6′. From these data, we verified the presence of one and two caffeoyl groups for compounds **4**–**5** and **6**–**9**, respectively. Additionally, all spectra showed multiplets in the region of δ 2.37–1.91 (m, 4H) corresponding to two methylene hydrogens, two signals in the regions δ 5.64–5.18 (m, 1H) and δ 4.37–4.04 (m, 1H), and a doublet of doublets in the region δ 5.12–3.97, which was assigned to hydrogen from methine groups bearing oxygen atoms. These signals and the ^13^C NMR data confirmed the presence of a quinic acid moiety in the structures. Compounds **5** and **9** exhibited an additional signal assigned to the presence of a methoxyl group at δ 3.60 (s, 3H) and δ 3.69 (s, 3H), respectively. Through HMBC correlations, we determined the positions of caffeoyl and methoxyl groups in the quinic acid moieties. Consequently, the compounds were identified through comparisons with literature data [30,31] as 5-*O*-caffeoylquinic acid (**4**; chlorogenic acid), 5-*O*-caffeoylquinic acid methyl ester (**5**), 3,4-di-*O*-caffeoylquinic acid (**6**), 3,5-di-*O*-caffeoylquinic acid (**7**), 4,5-di-*O*-caffeoylquinic acid (**8**), and 3,5-di-*O*-caffeoylquinic acid methyl ester (**9**) (Figure 2). Compound **10** was identified as caffeic acid through the standard addition of a commercial sample of the compound using HPLC-DAD-UV.

Compounds **11**–**13** showed five signals in the aromatic region in their ^1^H NMR spectra. Two doublets were found in the region δ 7.57–7.53 (d, *J* = 2.1 Hz, 1H) and at δ 6.82 (d, *J* = 9.0 Hz, 1H) and a doublet of doublets was found at 7.57–7.54 (dd, *J* = 9.0, 2.1 Hz, 1H), which were assigned to H-2′, H-5′, and H-6′, respectively, of the 3,4-dihydroxy B-ring of flavonoids. Two large singlets were observed in the regions δ 6.37–6.35 and δ 6.17–6.16, which were assigned to H-8 and H-6 from the A-ring of flavonoids. These data were corroborated by UV spectra, which showed absorption bands at 254, 269, and 360 nm characteristic of flavonols [27]. The absence of the signal of H-3 in the spectra, as well as the presence of an anomeric hydrogen at δ 5.51–5.32 (d, *J* = 7.3 Hz, 1H), confirmed the presence of quercetins-3-*O*-glycosides. For compounds **11** and **12**, additional doublets at δ 3.56 (d, *J* = 11.5 Hz, 2H) and at δ 0.91 (d, *J* = 5.6 Hz, 3H) were attributed to H-6″ of the glucose and rhamnose units, respectively. Compound 13 exhibited two doublets at δ 5.33 (d, *J* = 7.2 Hz, 1H) and at δ 0.98 (d, *J* = 6.1 Hz, 3H) and a broad singlet at δ 4.36 (sl, 1H), which were assigned to H-1″, H-6″′, and H-1″′ of the rutinoside (α-rhamnopyranosyl-(1→6)-β-glucopyranoside) unit. Compound **14** showed four doublets in the spectrum at δ 8.01 (d, *J* = 8.9 Hz, 2H) and δ 6.85 (d, *J* = 8.9 Hz, 2H)—which were respectively assigned to H-2′/H-6′ and H-3′/H-5′ of the *p*-hydroxylated B-ring of the flavonoid—and at δ 6.31 (d, *J* = 1.6 Hz, 1H) and δ 6.10 (d, *J* = 1.6 Hz, 1H), which were attributed to H-8 and H-6 of the kaempferol derivatives, respectively. Similarly to what was observed for compound 13, the presence of anomeric hydrogen at δ 5.21 (d, *J* = 7.6 Hz, 1H) and the signals at δ 4.39 (sl, 1H) and at δ 1.04 (d, *J* = 6.2 Hz, 3H) were attributed to the robinobioside moiety (α-rhamnopyranosyl-(1→6)-β-galactopyranoside). Finally, for compounds **11**–**14**, the multiplets in the region δ 3.90–3.00 were attributed to other sugar protons. The ^13^C NMR spectra of compounds **11**–**14** and a comparison with the literature data [30,31,32,33] confirmed the structures of the compounds as those of isoquercitrin (**11**; quercetin-3-*O*-β-glucopyranoside), quercitrin (**12**; quercetin-3-*O*-β-rhamnopyranoside), rutin (**13**; quercetin-3-*O*-α-rhamnopyranosyl-(1→6)-β-glucopyranoside or quercetin-3-*O*-rutinoside), and biorobin (**14**; kaempferol-3-*O*-α-rhamnopyranosyl-(1→6)-β-galactopyranoside or kaempferol-3-*O*-robinobioside) (Figure 2).

Chromatographic fractionation of the BuOH fraction afforded the compounds **4** and **5**, previously identified in the ethyl acetate phase; additionally, compound **15** was isolated. The UV spectrum showed characteristic absorptions of flavones at 272 and 326 nm [27]. The ^1^H NMR spectrum showed two doublets at δ 8.08 (d, *J* = 8.5 Hz, 2H) and δ 6.93 (d, *J* = 8.5 Hz, 2H) and a singlet at δ 6.90 (s, 1H), suggesting an apigenin derivative. No additional aromatic signals were observed in the spectrum; therefore, the presence of a totally substituted A-ring was inferred. Moreover, two doublets at δ 4.91 (d, *J* = 10.1 Hz, 1H) and δ 4.76 (d, *J* = 10.1 Hz, 1H), as well as multiplets in the region δ 3.90–3.20, suggested the presence of a di-C-glycosyl-apigenin. The ^13^C NMR data and a comparison with the literature data [34] confirmed the compound as vicenin-2 (**15**; 6,8-di-C-β-glucopyranosyl-apigenin).

### 2.2. Antiradical Properties of the Extract, Fractions, and Compounds

The samples (ethanol extract of *Baccharis sphenophylla*, three partition fractions, and isolated compounds) were evaluated in relation to their antiradical capacities using DPPH and ABTS assays. Table 1 shows the results obtained for the extract and partition fractions.

Although the differences in the values of the antiradical capacities were observed with both methods, the trends for the samples were internally similar (Table 1). Values obtained with the ABTS assay were higher than those obtained with the DPPH assay. Additionally, the antiradical capacity of the ethyl acetate fraction of *B. sphenophylla* was higher than the ethanol crude extract, as well as the other fractions from this extract.

The isolated compounds were evaluated in both assays, and the results are shown in Table 2. The *ent*-kaurenoic acid (**1**) was not able to scavenge the radicals used in either assay; therefore, the IC_50_ was not determined (value higher than 200 μmol.L^−1^). The same pattern of response obtained with the mixtures was observed for the isolated compounds; i.e., the values for the antiradical capacity in the ABTS assay were higher than those obtained with the DPPH assay.

When analyzing the isolated compounds, flavonoids (**2**–**3**, **11**–**15**) showed lower antiradical activities. Among these compounds, the flavonols (**11**–**14**) were more active than the flavones (**2**–**3**, **15**). On the other hand, chlorogenic acid derivatives (**4**–**10**) were more active than flavonoids and Trolox, showing a greater ability to trap radicals.

## 3. Discussion

This study revealed for the first time the phytochemical composition of the polar extract of *Baccharis sphenophylla*. Fifteen compounds were isolated and identified and, among them, fourteen are described for the first time for this species. These compounds were diterpenoids, flavonoids, and chlorogenic acid derivatives, which are natural products frequently found in *Baccharis* species [4,5].

The antiradical capacities of different polar fractions of the plant were evaluated using two assays. The obtained results showed that the antiradical capacities were different in the two assays for the same samples. This fact can be explained by the reactivity of both the generated radicals. The stable free radical DPPH only interacted with more reactive antiradical compounds (constituents with low reactivity found in the mixture are would probably not have been detected by this assay), while the ABTS free radical was able to react with these compounds. Therefore, the differences in the capacity values determined in these assays can be attributed to the presence of low-reactivity antiradical compounds in the samples. A similar pattern of response was observed with the hydroalcoholic crude extracts of *Baccharis burchellii* and *B. crispa* analyzed with these assays [35]. The higher antiradical capacity showed by the ethyl acetate fraction can be explained by its chemical composition. This sample was mainly composed of flavonoids and chlorogenic acid derivatives, natural products known for their antiradical activity [13,36].

When analyzing the flavonoids (compounds **2**–**3** and **11**–**15**), flavonols (**11**–**14**) had a greater ability to scavenge the radicals than the flavones (**2**–**3**, **15**). The isolated flavones were apigenin (compounds **2** and **15**) or luteolin (compound **3**) derivatives. The first do not contain the catechol group in their structures, which further decreases their antiradical capacities [37]. Additionally, compounds **2**–**3** had methoxylated structures, contributing to the decrease in their antiradical capacity. However, the oxygenation of C-3 found in the flavonols contributed to the higher activity observed for these samples. Among the flavonols, those with the catechol group in the B ring (compounds **11**–**13**) had the highest antiradical capacities. For flavonoids, the structural requirements to achieve better radical scavenging activities are: (i) the presence of a catechol group in ring B, which has better electron-donating properties and is a radical target; (ii) a 2,3-double bond conjugated with the 4-oxo group, which is responsible for electron delocalization; and (iii) the presence of a 3-hydroxyl group in the heterocyclic ring, which also increases radical scavenging activity [37]. The flavonoids isolated from the leaves of *B. sphenophylla* seemed to follow these general trends. Lastly, the different glycosides found in the structures of flavonols (**11**–**14**) had no apparent effects on the antiradical activity.

Chlorogenic acid derivatives are already known for their potent antioxidant capacity because they have a greater capacity to stabilize radicals due to the presence of a catechol group in their structures. Comparing the chlorogenic acid derivatives containing two caffeoyl groups bonded in the quinic acid (compounds **6**–**9**) with those that showed only one caffeoyl group in the structure (compounds **4** and **5**), the di-caffeoylquinic acids showed higher antiradical activity. This fact can be justified by the presence of an additional caffeoyl group esterified in the quinic acid (Table 2). The most active compounds were the 3,4-di-*O*-caffeoylquinic acid (**6**) and 4,5-di-*O*-caffeoylquinic acid (**8**), both of them having the caffeoyl group esterified in the C-4 position of the quinic acid. In the literature, the antiradical activity of di-caffeoylquinic acids against DPPH radicals has been analyzed, showing IC_50_ values compatible with those reported in this work [30,38]. When comparing the antiradical activity of methyl esters with their respective acids, it was possible to detect an increase in the activity. The higher lipophilicity of methyl esters could have been associated with slightly superior stabilization of both radicals. In summary, among the chlorogenic acids, esterification at the C-4 position seemed more relevant for antiradical activity, additional caffeoyl groups in the structures increased the antiradical activity, and methyl esters derivatives showed a higher capacity for trapping radicals than their respective acids.

## 4. Materials and Methods

### 4.1. General Experimental Procedures

Column chromatography (CC) was performed with a Sephadex LH-20 (GE Healthcare, Chicago, IL, USA) or silica gel 60 (Merck, Darmstadt, Germany). HPLC-grade solvents with the T.J. Baker trademark were used for the HPLC chromatography analyses. Analytical HPLC-DAD-UV analyses were carried out with an Agilent 1260 system (1260 Infinity LC system, Agilent Technologies, La Jolla, CA, USA) equipped with an ultraviolet spectrum scanning detector using an arrangement of photodiodes with a 60 mm flow cell. A Zorbax Eclipse plus a reverse phase C_18_ column (4.6 mm × 150 mm, 3.5 μm, Agilent, La Jolla, CA, USA) was used as the stationary phase, and a flow rate of 1.0 mL·min^−1^ was employed for analysis on an analytical scale with the column temperature set to 45 °C. The injection volume of the sample was 3 μL and the sample was dissolved in methanol at a concentration of 1 mg.mL^−1^. For the separation of compounds, an Agilent 1200 semi-preparative chromatography system (1200 LC system, Agilent Technologies, La Jolla, CA, USA) was used with a C_18_ Zorbax Eclipse plus an LC-18 column (25 cm × 10 mm, 5 μm, Agilent, La Jolla, CA, USA), a flow rate of 4.176 mL·min^−1^ for solvents, and a column temperature of 45 °C. The injection volume for the sample was 200 μL and the sample was dissolved in methanol at a concentration of 100 g.L^−1^. Both scales (analytical and semi-preparative) were employed as solvents: A—milli-Q water acidified with 0.1% acetic acid (*v*/*v*) and B—acetonitrile (ACN).

The nuclear magnetic resonance (NMR) spectra of hydrogen-1 (^1^H NMR) and carbon-13 (^13^C NMR) were recorded on a Bruker Avance III 300 Fourier-transform spectrometer (Bruker, Bremen, Germany) equipped with a 5 mm probe and operating at 300.11 MHz for ^1^H NMR and 75.5 MHz for ^13^C NMR at the Institute of Chemistry of the University of São Paulo. Standard pulse sequences from the Bruker TopspinTM (Bruker, Bremen, Germany) library were used for two-dimensional spectra. Gradient-enhanced sequences were used for the heteronuclear two-dimensional experiments. Chloroform-D, dimethylsulfoxide-d_6_, or methanol-d_4_ from Sigma-Aldrich (St. Louis, MO, USA) were used as solvents, with all shifts referred to internal TMS. All NMR spectra were processed using Mestrelab MestreNOVA software (version 12.0.0.20080).

### 4.2. Plant Material

Leaves of *Baccharis sphenophylla* Dusén ex Malme (Asteraceae) were collected from Campos do Jordão, São Paulo, Brazil, on 21 January 2016 (−22°77′33″ S; −45°56′25″ W). The plant was identified by O. A. Fávero, and a voucher specimen (Fávero, OA.—CJ177) was deposited at the Herbarium Embrapa Clima Temperado (ECT 0003699) and registered with the Sistema Nacional de Gestão do Patrimônio Genético e do Conhecimento Tradicional Associado (SisGen # A47125D).

### 4.3. Extraction and Isolation of Compounds

Dried leaves of *B. sphenophylla* (384 g) were powdered and extracts obtained with hexane and then with ethanol (EtOH). EtOH extract (65.2 g) was resuspended in EtOH:H_2_O (1:1) and partitioned successively with dichloromethane (DCM), ethyl acetate (EtOAc), and *n*-butanol (BuOH).

The dichloromethane fraction (DCMF, 1.5 g) was subjected to fractionation in the chromatography column (Silica gel 60) eluted with hexane, hexane:ethyl acetate (9:1, 7:3, 1:1, 3:7, and 0:1), and ethyl acetate:methanol (7:3, 1:1, 3:7, and 0:1), resulting in five groups after TLC analysis. DCMF-2 (575.3 mg) resulted in the *ent*-kaurenoic acid (**1**). DCMF-3 (209.6 mg) and DCMF-4 (103.2 mg) were purified with semi-preparative HPLC (solvent A: water acidified with 0.1% acetic acid; solvent B: acetonitrile; linear gradient (% B): 0–10 min—10–25%, 10–30 min—25–50%), giving rise to hispidulin (**2**; 41.2 mg) and eupafolin (**3**; 193.5 mg).

The EtOAc fraction (1.3 g) was subjected to Sephadex LH-20 column chromatography and eluted with methanol to produce six groups (A–F). Groups B (80 mg) and C (59 mg) were subjected to separation using semi-preparative HPLC (same conditions as DCMF; method: 0–3 min: 10 → 20% B; 3–7 min: 25% B) to produce caffeic acid (**10**, 15.3 mg), chlorogenic acid (**4**, 70.5 mg), and 5-*O*-caffeoylquinic acid methyl ester (**5**, 21.3 mg). Group D (103 mg) was subjected to semi-preparative HPLC (method: 0–3 min: 10 → 20% B; 3–7 min: 20% B; 7–8 min: 20 → 25% B; 8–12 min: 25% B; 12–15 min: 25 → 40% B), yielding isoquercitrin (**11**, 12.4 mg, quercetin-3-*O*-β-glucopyranoside), quercitrin (**12**, 11.3 mg, quercetin-3-*O*-α-rhamnopyranoside), rutin (**13**, 29.3 mg, quercetin-3-*O*-β-(6″-*O*-α-rhamnosyl)-glucopyranoside), and biorobin (**14**, 9.8 mg, kaempferol-3-*O*-β-(6″-*O*-α-rhamnosyl)-galactopyranoside). Group E (602 mg) was subjected to semi-preparative HPLC (method: 0–3 min: 10 → 20% B; 3–7 min: 20% B; 7–8 min: 20 → 25% B; 8–12 min: 25% B; 12–17 min: 25 → 50% B; 17–22 min: 50 → 100% B; 22–22.5 min: 100% B) to yield 3,4-di-*O*-caffeoylquinic acid (**6**, 93.5 mg), 3,5-di-*O*-caffeoylquinic acid (**7**, 277.3 mg), and 4,5-di-*O*-caffeoylquinic acid (**8**, 135.9 mg). The same elution condition used for group E (115 mg) was used to subject group F to semi-preparative HPLC. Group F afforded 3,5-di-*O*-caffeoylquinic acid methyl ester (**9**, 33.7 mg).

The *n*-butanol fraction (BuF, 1.0 g) was subjected to Sephadex LH-20 column chromatography and eluted with methanol, giving rise to four groups. BuF-1 and BuF-2 showed the presence of compounds **4**, **5**, and **10**, previously isolated and identified in the ethyl acetate fraction. BuF-3 (253.1 mg) yielded vicenin-2 (**15**, 6,8-di-*C*-β-glucopyranosyl-apigenin). The NMR data for the characterization of the isolated compounds are shown in the Supplementary Material.

### 4.4. Antioxidant Assays

Antioxidant assays were performed using a microplate reader, BioTek^®^ Synergy™ H1 (Agilent Technologies, Santa Clara, CA, USA), with 96-well microplates. Methanol was used as a solvent for the dilutions of the fractions, isolated compounds, Trolox (standard), and negative control. The data analyses were carried out using the software Statistica version 11 (StatSoft, Tulsa, OK, USA).

#### 4.4.1. DPPH Radical Scavenging Assay

The DPPH radical scavenging assay was carried out as described in the literature [35]. Briefly, 3.5 to 3.9 mg of DPPH was dissolved in 50 mL of methanol to prepare the DPPH solution. The exact concentration of the DPPH solution was determined spectrophotometrically with the maximum absorbance at 515 nm (εDPPH = 1.25 × 10^4^ L.mol^−1^.cm^−1^). The Trolox antiradical solution was prepared with 1.25 mg of the compound dissolved in 2.5 mL of methanol. The prepared solutions were placed in an ultrasonic homogenizer for 5 min to ensure complete solubilization.

Analyses were performed with a microplate reader for absorbance with an optical path of 5 mm and a total volume of 220 μL. Measurements were initiated with the addition of 200 μL DPPH to 20 μL of the sample solution (extract or pure compound with antiradical activity). The kinetics of the reaction was measured from the absorbance of the DPPH solution at 515 nm. All kinetic tests were performed in triplicate with independent measurements, and the results were analyzed and represented as the mean ± standard deviation in the program Origin Pro 8.5 to obtain the kinetic curves.

The variation in absorbance (ΔAbs.) between T0 and T50 (AbsTinitial—AbsTfinal) showed a linear correlation with the antiradical concentration. In order to calculate the antiradical activity of the fractions and pure compounds, the angular coefficients (α) of the antiradical (A) and Trolox (T) standard deviations were used as a function of the absorbance variation, making it possible to obtain the corresponding antiradical capacity as a percentage of Trolox (%Tx) using Equation (1) [35].

% Tx = (αA/αT) × 100(1)

Using Equation (2), the percentage of antiradical activity was calculated. The negative control was prepared with 200 μL of DPPH and 20 μL of methanol, the blank was prepared with 20 μL of the sample and 200 μL of methanol, and the sample was prepared with 20 μL of the solution of the fraction or compound and 200 μL of DPPH. The 50% inhibitory concentration (IC_50_) of each compound was obtained from the equation for the straight line of the concentration graph using the percentage of antiradical activity.

AA% = 100 − {[(ABS_SAMPLE_ − ABS_BLANK_) × 100]/ABS_NEGATIVE_} (2)

#### 4.4.2. ABTS Assay

The ABTS assay was performed as described in the literature [35,39]. Briefly, the stock solution of ABTS (7.0 × 10^−3^ mol.L^−1^, 2,2′-azobis-(3-ethyl-benzothiazoline-6-sulfonic acid)) was prepared by dissolving ABTS (19.2 mg) in deionized water (5.0 mL). This solution was stored at 4 °C and protected from light for up to 30 days. The ABTS^+•^ radical was prepared and 1 mL of 7 mM ABTS solution was mixed with 17.6 μL of 140 mM potassium persulfate and protected from light for 16 h. The final concentration was determined spectrophotometrically (ε_734 nm_ = 1.5 × 10^4^ L.mol^−1^.cm^−1^). After this period, 1 mL of the ABTS^+•^ solution was diluted in 40 mL of methanol (absorbance ~ 1; pH 6.7). Aliquots of 20 μL of the samples (compounds (250 μg.mL^−1^), negative control, and Trolox (80–400 μM)) and 280 μL of ABTS^+•^ solution were transferred into each well of the microplate. The absorbance at 734 nm was monitored for 30 min. After 20 min of incubation, the absorbance was measured at 734 nm [35,39].

The software Statistica version 11 (StatSoft, USA) was used for the analysis of variance (ANOVA) with a level of significance of *p* < 0.05. Tuckey’s method was used to check for significant differences between the groups (α = 95%).

## 5. Conclusions

This paper described fifteen compounds obtained from polar extracts of *B. sphenophylla*. Additionally, the extracts and isolated compounds (flavonoids and chlorogenic acids) were evaluated as sources of antiradical constituents. These metabolites showed higher radical scavenging activity in the investigated assays, confirming the *Baccharis* species as an important source of phenolic compounds with antiradical properties.

## Figures and Tables

**Table 1:** Antiradical capacities of crude extract and partition fractions obtained from *B. sphenophylla*.

	% Trolox ^1^	
Samples ^2^	DPPH	ABTS
EtOH extract	33.8 ± 0.1 ^b^	36.1 ± 0.3 ^b^
DCM fraction	20.3 ± 0.2 ^a^	22.1 ± 0.4 ^a^
EtOAc fraction	88.9 ± 0.2 ^c^	90.4 ± 0.1 ^c^
BuOH fraction	34.7 ± 0.1 ^b^	40.8 ± 0.2 ^b^

^1^ % Trolox is expressed in mg.L^−1^; ^2^ EtOH extract: ethanol extract; DCM, EtOAc, and BuOH fractions: dichloromethane, ethyl acetate, and *n*-butanol fractions. Different letters indicate statistical difference (*p* < 0.05).

**Table 2:** Antiradical capacities of compounds isolated from *B. sphenophylla*.

		IC_50_ (µmol.L^−1^)	
Compounds ^1^	Source ^2^	DPPH	ABTS
(**2**) Hispidulin	DCM	119.7 ± 10.2 ^f^	103.1 ± 8.7 ^f^
(**3**) Eupafolin	DCM	89.8 ± 2.1 ^e^	85.1 ± 0.9 ^e^
(**4**) Chlorogenic acid	EtOAc	29.1 ± 1.3 ^b^	20.4 ± 0.4 ^b^
(**5**) Chlorogenic acid methyl ester	EtOAc	27.1 ± 2.4 ^b^	18.3 ± 0.2 ^a,b^
(**6**) 3,4-di-*O*-Caffeoylquinic acid	EtOAc	14.7 ± 1.3 ^a^	10.9 ± 1.1 ^a^
(**7**) 3,5-di-*O*-Caffeoylquinic acid	EtOAc	17.2 ± 1.1 ^a,b^	15.4 ± 1.5 ^a,b^
(**8**) 4,5-di-*O*-Caffeoylquinic acid	EtOAc	14.1 ± 0.5 ^a^	10.1 ± 0.5 ^a^
(**9**) 3,5-di-*O*-Caffeoylquinic acid methyl ester	EtOAc	15.7 ± 0.8 ^a^	10.7 ± 0.4 ^a^
(**10**) Caffeic acid	EtOAc	25.3 ± 1.2 ^b^	22.1 ± 1.2 ^b^
(**11**) Isoquercitrin	EtOAc	34.3 ± 1.9 ^b,c^	31.3 ± 0.9 ^b,c^
(**12**) Quercitrin	EtOAc	34.9 ± 1.3 ^b,c^	31.1 ± 1.2 ^b,c^
(**13**) Rutin	EtOAc	35.5 ± 1.5 ^b,c^	33.5 ± 0.8 ^b,c^
(**14**) Biorobin	EtOAc	69.9 ± 0.8 ^d^	62.7 ± 0.7 ^d^
(**15**) Vicenin-2	*n*-BuOH	90.3 ± 1.1 ^e^	85.8 ± 2.1 ^e^
Trolox	standard	45.5 ± 1.5	40.1 ± 1.1

^1^ The *ent*-kaurenoic acid (**1**) isolated from the DCM fraction was not able to scavenge the radicals used in either assay; ^2^ this column describes the source from which the compounds were isolated. DCM, EtOAc, and n-BuOH are, respectively, the dichloromethane, ethyl acetate, and *n*-butanol fractions. Different letters indicate statistical difference (*p* < 0.05).

## Data Availability

All data generated during this study are included in this article.

## Supplementary Materials

The following supporting information can be downloaded at: https://www.mdpi.com/article/10.3390/plants12061262/s1, NMR data used for characterization of isolated compounds.
