# Supplementary material for: Chemical Constituents from Leaves of Baccharis sphenophylla (Asteraceae) and Their Antioxidant Effects
Source: Plants (Basel). 2023 Mar 10;12(6):1262. doi: 10.3390/plants12061262 (PMC10051307; doi:10.3390/plants12061262)
Supplement: Supplementary file 1 [file plants-12-01262-s001.zip › plants-2197480-supplementary.pdf]

Supplementary Materials

Chemical Constituents from Leaves of *Baccharis sphenophylla* (Asteraceae) and Their Antioxidant Effects

Marcela H. Retamozo [1], Christian C. Silva [1], Cinthia I. Tamayose [1], Juliana C. S. Carvalho [1], Paulete Romoff [2], Oriana A. Fávero [3] and Marcelo J. P. Ferreira [1,*]

* Correspondence: marcelopena@ib.usp.br; Tel.: +55-11-3091-7546

NMR data for characterization of the isolated compounds

ent-Kaurenoic acid (**1**): ^{1}H NMR (300 MHz, $CDCl_3$) δ_H: 0.80 (1H, m, H-1), 1.9-1.8 (4H, m, H-1, H-2, H-6), 1.6-1.4 (7H, m, H-2, H-7, H-11, H-12), 1.03 (1H, m, H-3), 2.15 (1H, m, H-3), 1.2-1.1 (3H, m, H-5, H-9, H-14), 2.65 (1H, sl, H-13), 2.00 (1H, m, H-14), 2.19 (2H, s, H-15), 4.81 (1H, sl, H-17), 4.76 (1H, sl, H-17), 0.97 (3H, s, H-18), 1.27 (3H, s, H-20). ^{13}C NMR (75.5 MHz, $CDCl_3$) δ_C: 40.7 (C-1), 19.1 (C-2), 37.8 (C-3), 43.7 (C-4), 57.0 (C-5), 21.8 (C-6), 41.3 (C-7), 44.2 (C-8), 55.1 (C-9), 39.6 (C-10), 18.4 (C-11), 33.1 (C-12), 43.8 (C-13), 39.7 (C-14), 49.0 (C-15), 155.9 (C-16), 103.0 (C-17), 29.0 (C-18), 183.8 (C-19), 15.6 (C-20).

Hispidulin (**2**): ^{1}H NMR (300 MHz, DMSO-d_6) δ_H: 6.73 (1H, s, H-3), 6.58 (1H, s, H-8), 7.89 (2H, d, *J*= 8.8 Hz, H-2′, H-6′), 6.92 (2H, d, *J*= 8.8 Hz, H-3′, H-5′), 3.75 (3H, s, OCH_3), 13.05 (s, OH-C_5). ^{13}C NMR (75.5 MHz, DMSO-d_6) δ_C: 164.3 (C-2), 102.8 (C-3), 182.5 (C-4), 153.2 (C-5), 131.7 (C-6), 157.7 (C-7), 94.7 (C-8), 152.8 (C-9), 104.4 (C-10), 121.6 (C-1′), 128.9 (C-2′, C-6′), 116.4 (C-3′, C-5′), 161.7 (C-4′), 60.6 (OCH_3).

Eupafolin (**3**): ^{1}H NMR (300 MHz, DMSO-d_6) δ_H: 6.66 (s, H-3), 6.58 (s, H-8), 7.40 (1H, d, *J*= 2.0 Hz, H-2′), 6.89 (1H, d, *J*= 8.0 Hz, H-5′), 7.42 (1H, dd, *J*= 8.0 and 2.0 Hz, H-6′), 3.75 (3H, s, OCH_3), 13.09 (s, OH-C_5). ^{13}C NMR (75.5 MHz, DMSO-d_6) δ_C: 164.2 (C-2), 102.9 (C-3), 182.3 (C-4), 153.1 (C-5), 131.8 (C-6), 158.3 (C-7), 94.5 (C-8), 152.8 (C-9), 104.2 (C-10), 121.8 (C-1′), 113.6 (C-2′), 146.2 (C-3′), 150.2 (C-4′), 116.4 (C-5′), 119.2 (C-6′), 60.3 (OCH_3).

5-*O*-Caffeoylquinic acid (**4**): ^{1}H NMR (300 MHz, CD_3OD) δ_H: 1.90-2.10 (4H, m, H-2, H-6), 5.17 (1H, m, H-3), 3.70 (1H, dd, *J*= 3.3, 7.5 Hz, H-4), 4.08 (1H, dd, *J*= 3.2, 6.9 Hz, H-5), 7.13 (d, *J*= 1.8 Hz, H-2′), 6.84 (d, *J*= 8.4 Hz, H-5′), 7.03 (dd, *J*= 1.8, 8.4 Hz, H-6′), 7.58 (d, *J*= 16.0 Hz, H-7′), 6.34 (d, *J*= 16.0 Hz, H-8′). ^{13}C NMR (75.5 MHz, CD_3OD) δ_C: 73.5 (C-1), 37.4 (C-2), 72.5 (C-3), 72.9 (C-4), 70.1 (C-5), 38.6 (C-6), 176.3 (C-7), 127.6 (C-1′), 115.4 (C-2′), 147.1 (C-3′), 149.7 (C-4′), 116.9 (C-5′), 123.3 (C-6′), 147.5 (C-7′), 115.5 (C-8′), 168.5 (C-9′).

5-*O*-Caffeoylquinic acid methyl ester (**5**): ^{1}H NMR (CD_3OD, 300 MHz): δ 1.91–2.08 (4H, m, H-2, H-6), 5.18 (1H, m, H-3), 3.63 (1H, dd, *J* = 3.1, 7.4 Hz, H-4), 4.04 (1H, dd, *J* = 3.3, 6.7 Hz, H-5), 7.06 (1H, d, *J* = 1.9 Hz, H-2′), 6.80 (1H, d, *J* = 8.1 Hz, H-5′), 6.97 (1H, dd, *J* = 1.9, 8.1 Hz, H-6′), 7.55 (1H, d, *J* = 15.9 Hz, H-7′), 6.24 (1H, d, *J* = 15.9 Hz, H-8′); 3.60 (3H, s, $COOCH_3$); ^{13}C NMR (CD_3OD, 75.5 MHz) δ_C: 75.9 (C-1), 37.9 (C-2), 72.3 (C-3), 72.6 (C-4), 70.4 (C-5), 38.2 (C-6), 175.6 (C-7), 127.8 (C-1′), 115.2 (C-2′), 147.0 (C-3′), 149.9 (C-4′), 116.7 (C-5′), 123.0 (C-6′), 147.4 (C-7′), 115.3 (C-8′), 168.4 (C-9′), 53.1 (OCH_3).

3,4-di-*O*-Caffeoylquinic acid (**6**): ^{1}H NMR (300 MHz, CD_3OD) δ_H: 2.12 (2H, m, H-2), 5.64 (m, H-3), 5.12 (dd, *J*= 2.9, 9.1 Hz, H-4), 4.37 (sl, H-5), 2.27 (2H, m, H-6), 7.01 / 7.02 (d, *J*= 2.0 Hz, H-2′ / H-2′′), 6.74 / 6.75 (d, *J*= 8.2 Hz, H-5′ / H-5′′), 6.90 / 6.92 (dd, *J*= 2.0, 8.2 Hz, H-6′ / H-6′′), 7.52 / 7.60 (d, *J*= 15.9 Hz, H-7′ / H-7′′), 6.19 / 6.29 (d, *J*= 15.9 Hz, H-8′ / H-8′′). ^{13}C NMR (75.5 MHz, CD_3OD) δ_C: 76.0 (C-1), 39.6 (C-2), 69.2 (C-3), 76.0 (C-4), 69.7 (C-5), 38.5 (C-6), 176.0 (C-7), 127.7 (C-1′), 115.3 (C-2′), 146.9 (C-3′), 149.8 (C-4′), 123.3 (C-5′), 116.6 (C-6′), 147.7 (C-7′), 115.3 (C-8′), 168.7 (C-9′), 127.8 (C-1′′), 114.9 (C-2′′), 146.9 (C-3′′), 149.8 (C-4′′), 123.3 (C-5′′), 116.6 (C-6′′), 147.8 (C-7′′), 114.8 (C-8′′), 168.4 (C-9′′).

3,5-di-*O*-Caffeoylquinic acid (**7**): ^{1}H NMR (300 MHz, CD_3OD) δ_H: 2.20 (2H, m, H-2), 5.38 (m, H-3), 3.97 (dd, *J*= 3.2, 7.8 Hz, H-4), 5.42 (sl, H-5), 2.34 (2H, sl, H-6), 7.07 (2H, sl, H-2′, H-2′′), 6.78 (2H, d, *J*= 8.2 Hz, H-5′, H-5′′), 6.97 (2H, dl, *J*= 8.2 Hz, H-6′, H-6′′), 7.58 / 7.61 (d, *J*= 15.9 Hz, H-7′ / H-7′′), 6.27 / 6.36 (d, *J*= 15.9 Hz, H-8′ / H-8′′). ^{13}C NMR (75.5 MHz, CD_3OD) δ_C: 74.7 (C-1), 38.2 (C-2), 72.2 (C-3), 71.0 (C-4), 72.8 (C-5), 36.1 (C-6), 177.7 (C-7), 127.8 (C-1′), 115.3 (C-2′), 146.8 (C-3′), 149.6 (C-4′), 123.1 (C-5′), 116.5 (C-6′), 147.1 (C-7′), 115.7 (C-8′), 169.0 (C-9′), 128.0 (C-1′′), 115.2 (C-2′′), 146.5 (C-3′′), 149.5 (C-4′′), 123.0 (C-5′′), 116.5 (C-6′′), 147.3 (C-7′′), 115.2 (C-8′′), 168.4 (C-9′′).

4,5-di-*O*-Caffeoylquinic acid (**8**): ^{1}H NMR (300 MHz, CD_3OD) δ_H: 2.12 (m, H-2 / H-6), 2.20 (sl, H-2), 4.36 (m, H-3), 5.01 (dd, *J*= 3.2, 8.8 Hz, H-4), 5.64 (sl, H-5), 2.37 (sl, H-6), 7.02 / 7.04 (d, *J*= 1.8 Hz, H-2′ / H-2′′), 6.73 / 6.77 (d, *J*= 8.2 Hz, H-5′ / H-5′′), 6.88 / 6.91 (dd, *J*= 1.8, 8.2 Hz, H-6′ / H-6′′), 7.55 / 7.57 (d, *J*= 15.9 Hz, H-7′ / H-7′′), 6.26 / 6.28 (d, *J*= 15.9 Hz, H-8′ / H-8′′). ^{13}C NMR (75.5 MHz, CD_3OD) δ_C: 76.6 (C-1), 41.9 (C-2), 65.9 (C-3), 75.2 (C-4), 70.3 (C-5), 37.0 (C-6), 178.2 (C-7), 127.9 (C-1′), 115.2 (C-2′), 146.9 (C-3′), 149.8 (C-4′), 123.3 (C-5′), 116.6 (C-6′), 147.2 (C-7′), 115.3 (C-8′), 168.8 (C-9′), 127.8 (C-1′′), 115.1 (C-2′′), 146.9 (C-3′′), 149.8 (C-4′′), 123.1 (C-5′′), 116.5 (C-6′′), 146.2 (C-7′′), 115.0 (C-8′′), 168.7 (C-9′′).

3,5-di-*O*-Caffeoylquinic acid methyl ester (**9**): ^{1}H NMR (300 MHz, CD_3OD) δ_H: 2.16 (2H, m, H-2), 5.30 (m, H-3), 3.98 (dd, *J*= 3.2, 6.5 Hz, H-4), 5.40 (m, H-5), 2.32 (2H, m, H-6), 7.06 / 7.07 (d, *J*= 1.9 Hz, H-2′ / H-2′′), 6.78 / 6.79 (d, *J*= 8.2 Hz, H-5′ / H-5′′), 6.97 (2H, dd, *J*= 1.8, 8.2 Hz, H-6′, H-6′′), 7.55 / 7.62 (d, *J*= 15.9 Hz, H-7′ / H-7′′), 6.22 / 6.34 (d, *J*= 15.9 Hz, H-8′ / H-8′′), 3.69 (3H, s, OCH_3). ^{13}C NMR (75.5 MHz, CD_3OD) δ_C: 74.5 (C-1), 35.5 (C-2), 71.8 (C-3), 69.6 (C-4), 72.1 (C-5), 36.5 (C-6), 175.5 (C-7), 127.7 (C-1′), 114.7 (C-2′), 146.7 (C-3′), 149.4 (C-4′), 116.4 (C-5′), 123.9 (C-6′), 147.0 (C-7′), 114.9 (C-8′), 167.8 (C-9′), 127.5 (C-1′′), 114.9 (C-2′′), 146.6 (C-3′′), 149.6 (C-4′′), 116.5 (C-5′′), 123.9 (C-6′′), 147.7 (C-7′′), 115.3 (C-8′′), 168.6 (C-9′′), 52.9 (OCH_3).

Caffeic acid (**10**): this compound was identified through co-injection of the commercial standard of the sample.

Quercetin 3-*O*-β-glucopyranoside (**11**): ^{1}H NMR (300 MHz, DMSO-d_6) δ_H: 6.17 (1H, sl, H-6), 6.37 (1H, sl, H-8), 7.57 (1H, d, *J* = 2.1 Hz, H-2′), 6.82 (1H, d, *J* = 9.0 Hz, H-5′), 7.57 (1H, dd, *J* = 2.1, 9.0 Hz, H-6′), 5.45 (1H, d, *J* = 7.3 Hz, H-1″), 3.90 – 3.00 (4H, m, H-2′′ to H-5′′), 3.56 (2H, d, *J*= 11.5 Hz, H-6′′); ^{13}C NMR (75.5 MHz, DMSO-d_6) δ_C: 156.9 (C-2), 133.9 (C-3), 177.9 (C-4), 161.7 (C-5), 99.4 (C-6), 165.2 (C-7), 94.2 (C-8), 156.7 (C-9), 104.1 (C-10), 122.3 (C-1′), 115.8 (C-2′), 145.5 (C-3′), 148.7 (C-4′), 116.5 (C-5′), 121.7 (C-6′), 101.5 (C-1″), 74.5 (C-2″), 77.0 (C-3″), 70.4 (C-4″), 78.1 (C-5″), 61.5 (C-6″).

Quercetin 3-*O*-α-rhamnopyranoside (**12**): ^{1}H NMR (300 MHz, DMSO-d_6) δ_H: 6.16 (1H, sl, H-6), 6.35 (1H, sl, H-8), 7.55 (1H, d, *J* = 2.1 Hz, H-2′), 6.82 (1H, d, *J* = 9.0 Hz, H-5′), 7.55 (1H, dd, *J* = 2.1, 9.0 Hz, H-6′), 5.32 (1H, d, *J* = 7.3 Hz, H-1″), 3.90 – 3.00 (4H, m, H-2′′ to H-5′′), 0.91 (3H, d, *J*= 5.6 Hz, H-6′′); ^{13}C NMR (75.5 MHz, DMSO-d_6) δ_C: 158.1 (C-2), 134.9 (C-3), 177.9 (C-4), 163.1 (C-5), 99.5 (C-6), 166.0 (C-7), 94.5 (C-8), 159.1 (C-9), 105.6 (C-10), 123.3 (C-1′), 116.4 (C-2′), 145.7 (C-3′), 149.5 (C-4′), 117.7 (C-5′), 123.0 (C-6′), 102.5 (C-1″), 71.4 (C-2″), 70.9 (C-3″), 71.5 (C-4″), 68.5 (C-5″), 18.2 (C-6″).

Quercetin 3-*O*-β-(6′′-*O*-α-rhamnosyl)-glucopyranoside (**13**): ^{1}H NMR (300 MHz, DMSO-d_6) δ_H: 6.17 (1H, sl, H-6), 6.35 (1H, sl, H-8), 7.53 (1H, d, *J* = 2.1 Hz, H-2′), 6.82 (1H, d, *J* = 9.0 Hz, H-5′), 7.54 (1H, dd, *J* = 2.1, 9.0 Hz, H-6′), 5.33 (1H, d, *J* = 7.2 Hz, H-1″), 3.90 – 3.00 (6H, m, H-2′′ to H-6′′), 4.35 (1H, sl, H-1′′′), 3.90 – 3.00 (4H, m, H-2′′′ to H-5′′′), 0.98 (3H, d, *J*= 6.1 Hz, H-6′′′); ^{13}C NMR (75.5 MHz, DMSO-d_6) δ_C: 157.2 (C-2), 134.1 (C-3), 177.9 (C-4), 161.6 (C-5), 99.5 (C-6), 165.6 (C-7), 94.4 (C-8), 156.9 (C-9), 104.4 (C-10), 121.9 (C-1′), 115.9 (C-2′), 145.5 (C-3′), 149.7 (C-4′), 116.8 (C-5′), 121.5 (C-6′), 101.6 (C-1″), 74.6 (C-2″), 76.2 (C-3″), 70.5 (C-4″), 76.9 (C-5″), 67.7 (C-6″), 101.4 (C-1″), 71.2 (C-2″), 70.7 (C-3″), 71.7 (C-4″), 68.5 (C-5″), 18.4 (C-6″).

Kaempferol-3-*O*-β-(6′′-*O*-α-rhamnosyl)-galactopyranoside (**14**): ^{1}H NMR (300 MHz, DMSO-d_6) δ_H: 6.10 (1H, d, *J*= 1.6 Hz, H-6), 6.31 (1H, d, *J*= 1.6 Hz, H-8), 8.01 (2H, d, *J*= 8.9 Hz, H-2′, H-6′), 6.85 (2H, d, *J*= 8.9 Hz, H-

3′, H-5′), 5.21 (1H, d, *J*= 7.6 Hz, H-1′′), 4.39 (1H, sl, H-1′′′), 3.90-3.00 (10H, m, H-2′′ to H-6′′, and H-2′′′ to H-5′′′), 1.04 (3H, d, *J*= 6.2 Hz, H-6′′′). ^{13}C NMR (75.5 MHz, DMSO-d_6) δ_C: 156.8 (C-2), 133.8 (C-3), 177.6 (C-4), 161.5 (C-5), 100.2 (C-6), 168.5 (C-7), 94.7 (C-8), 157.2 (C-9), 103.0 (C-10), 121.5 (C-1′), 131.4 (C-2′, C-6′), 115.3 (C-3′, C-5′), 160.5 (C-4′), 103.5 (C-1″), 71.6 (C-2″), 73.5 (C-3″), 68.5 (C-4″), 74.2 (C-5″), 66.3 (C-6″), 100.2 (C-1″), 70.8 (C-2″), 71.1 (C-3″), 72.5 (C-4″), 68.9 (C-5″), 18.1 (C-6″).

6,8-di-*C*-β-Glucopyranosyl-apigenin (**15**): ^{1}H NMR (300 MHz, DMSO-d_6) δ_H: 6.90 (1H, s, H-3), 8.08 (2H, d, *J*= 8.5 Hz, H-2′, H-6′), 6.93 (2H, d, *J*= 8.5 Hz, H-3′, H-5′), 4.91 (1H, d, *J*= 10.1 Hz, H-1′′), 4.76 (1H, d, *J*= 10.1 Hz, H-1′′′), 3.90-3.20 (12H, m, H-2′′-H-6′′, and H-2′′′-H-6′′′). ^{13}C NMR (75.5 MHz, DMSO-d_6) δ_C: 164.6 (C-2), 103.2 (C-3), 182.9 (C-4), 159.3 (C-5), 108.3 (C-6), 161.9 (C-7), 106.0 (C-8), 155.8 (C-9), 103.1 (C-10), 122.2 (C-1′), 129.7 (C-2′, C-6′), 116.5 (C-3′, C-5′), 161.9 (C-4′), 72.6 (C-1″), 71.7 (C-2″), 79.6 (C-3″), 69.8 (C-4″), 82.6 (C-5″), 60.6 (C-6″), 74.8 (C-1″), 71.3 (C-2″), 79.6 (C-3″), 69.8 (C-4″), 81.6 (C-5″), 62.0 (C-6″).
